# Tranexamic acid in arthroscopic repair of rotator cuff tears in the shoulder: a randomized trial

**DOI:** 10.1590/1806-9282.20251543

**Published:** 2026-06-19

**Authors:** Aloísio Rosado, Tércio Maia Sousa, João Nogueira, Luciana Salles Branco-de-Almeida, Caio Marcio Barros de Oliveira, Marcelo Souza de Andrade, Ed Carlos Rey Moura, Almir Vieira Dibai, Plínio da Cunha Leal

**Affiliations:** 1 Federal University of Maranhão – São Luís (MA), Brazil.; 2 Federal University of Maranhão, Postgraduate Program in Physical Education – São Luís (MA), Brazil.; 3 Federal University of São Paulo, Postgraduate Program in Interdisciplinary Surgical Science – São Paulo (SP), Brazil.

**Keywords:** Arthroscopy, Tranexamic acid, Visual clarity

## Abstract

**OBJECTIVE:**

The aim of this study was to determine the effectiveness of tranexamic acid in improving intraoperative visual clarity in patients undergoing arthroscopic repair of rotator cuff tears.

**METHODS:**

This prospective, randomized, double-blinded clinical trial was conducted in Brazil, between July 2020 and September 2022, including patients who underwent surgery for arthroscopic repair of rotator cuff tears.

**RESULTS:**

In total, 20 patients were included in each group: control, oral, and intravenous. There were no statistically significant differences between groups regarding intraoperative interventions, including duration of surgery, anesthesia time, number of ablations, increase in irrigation pump pressure, or total volume of irrigating solution used. Likewise, no differences were observed between groups in the assessment of visual clarity, either subjective (initial, final, and median) or objective, although the groups that received tranexamic acid had slightly lower average turbidity compared to the control group.

**CONCLUSION:**

Tranexamic acid use did not demonstrate benefits concerning the objective/subjective visual clarity between the evaluated groups.

## INTRODUCTION

Rotator cuff tears (RCT) are common in orthopedic practice^1^, reaching 22% of patients over 65 years^2^. Surgery is a prevalent option in the treatment of arthroscopic rotator cuff repair, being the most commonly performed procedure^3^.

Knowing how to control bleeding during arthroscopic procedures on the shoulder is vital; several studies have evaluated the reduction of blood loss and hematoma occurrence using tranexamic acid (TXA). TXA is a synthetic analogue of lysine that acts as an antifibrinolytic agent. It competitively blocks lysine receptors on plasminogen, thereby preventing fibrin clot degradation (fibrinolysis) and reducing bleeding^4^.

Furthermore, the efficacy of TXA has been demonstrated in clinical trials, but their results still differ in visual clarity in shoulder arthroscopy^5^.

Thus, this trial aimed to determine the effectiveness of TXA in improving intraoperative visual clarity in patients undergoing arthroscopic repair of RCT.

## METHODS

### Study design

This prospective, randomized, double-blind clinical trial followed CONSORT guidelines and was conducted between July 1, 2020, and September 30, 2022, in São Luís, Maranhão, Brazil.

Patients 18 years old or older, after 3 months of ­physiotherapy without pain improvement, who underwent arthroscopic repair of a rotator cuff tear rated between grade 0 and grade 2 in the Goutallier classification after magnetic resonance ­imaging were included.

Patients with a history of coagulopathy, altered coagulogram, renal or hepatic disorders, uncontrolled hypertension (systolic pressure >180 mmHg), allergic to anesthetic agents, or with irreparable/massive RCT with other surgical procedure needs, like tenotomy of the long head of the biceps tendon, were excluded.

### Data collection

Patient sociodemographic information, including gender, age, race/ethnicity, comorbidities, weight, and BMI, was systematically collected. During surgery, anesthesia and surgery duration, number of radiofrequency ablation uses, pump pressure increases, and irrigation fluid volume were recorded.

The measurement of subjective visual clarity was performed by analyzing the three-grade visual clarity scoring system performed during the surgical procedure every 15 min. The surgeon assigned grade 1 when visibility was low, with active bleeding, making it impossible to proceed with the surgery; grade 2 when visibility was reasonable, with moderate bleeding that interfered with vision but did not preclude the surgery; and grade 3 when the professional had good visibility, with no apparent blood presence^6^.

Additionally, an objective visual clarity was quantified using computerized video analysis, in which surgical procedures were continuously recorded at 30 frames per second using a high-definition arthroscopic camera system. A custom image processing system was developed to generate histograms ­reflecting blood presence (calculated as the percentage of red pixels in the surgical field) and image sharpness (assessed through histogram analysis). This methodology provides a quantitative, ­observer-independent measure of visual clarity based on established principles of digital image processing. The images were independently analyzed by three surgeons to verify the correlation between the objective measures (% red pixels) and the subjective assessment of visual clarity.

### Sample size

Sample size calculation was performed using G*Power software (v.3.1.9.4). The primary outcome was visual clarity assessed by the three-grade subjective scale. Based on a 4.3% incidence of significant intraoperative bleeding^7^, an expected minimum clinically important difference of 0.5 points in the visual ­clarity scale between groups, a statistical power of 0.8 (80%), an alpha level of 0.05 (5%), and accounting for a 10% potential loss to follow-up, a minimum of 60 patients (20 per group) was required for adequate statistical power to detect clinically ­relevant differences.

### Randomization

Randomization was conducted using a 1:1:1 ratio with software, assigning patients to control (CG), oral TXA (OG), or intravenous TXA (IG) groups, and concealment was ensured with sealed, numbered envelopes opened just before surgery. Each group had 20 patients, and participants and investigators ­collecting study data were blinded to group assignment until analysis.

Patients in the control group (CG) received ascorbic acid tablets, 250 mg each, at a dose of 20 mg/kg, 2 h before the procedure, as the placebo, and intravenous saline solution 10 min before the skin incision, with the same volume as if they were to receive intravenous TXA at a dose of 15 mg/kg. Patients in the oral group (OG) received TXA tablets, 250 mg each, at 20 mg/kg, 2 h before the procedure. To comply with the double-blind nature, patients in this group also received intravenous saline 10 min before skin incision, with the same volume as if they were to receive intravenous TXA at a dose of 15 mg/kg. In the intravenous group (IG), TXA was administered at 15 mg/kg 10 min before the skin incision. Participants in this group also received ascorbic acid (250 mg) orally at a 20 mg/kg dose 2 h before the procedure.

The oral dosage of 20 mg/kg was chosen to account for the approximately 45% oral bioavailability of TXA, while the intravenous dosage of 15 mg/kg follows standard clinical protocols^8^. Although these dosages are not strictly pharmacologically equivalent, the oral administration 2 h preoperatively allows achievement of therapeutic plasma concentrations (>10 μg/mL) at the time of incision, with the prolonged elimination half-life (approximately 11 h oral vs. 2 h IV) maintaining adequate levels throughout the procedure^9^.

All drugs were administered by staff not involved in care or data collection. General anesthesia combined with an ultrasound-guided interscalene brachial plexus block was used for all patients. Anesthesia induction included IV midazolam, ropivacaine block, propofol, fentanyl, and rocuronium, maintained with sevoflurane. At the end of surgery, antiemetics and neuromuscular blockade reversal were administered, and anesthesia was discontinued when discharge criteria were met.

### Surgical procedure

Patients were positioned in lateral decubitus, with the affected arm under longitudinal traction using a foam cuff and velcro support attached to the surgical table. The same surgeon performed all surgical procedures. The technique followed a double-row repair with medial and lateral anchors: subacromial decompression was performed first, followed by preparation of the rotator cuff footprint on the greater tubercle and suture fixation. Only 0.9% saline was used for irrigation, with fluid pressure set at 50 mmHg and increased by 20 mmHg increments if bleeding compromised visualization. Systolic and diastolic blood pressure were maintained within 100–130 and 50–80 mmHg, respectively, using vasopressors or hypotensive medications as needed. Extubation occurred once patients were awake and stable; after surgery, they remained under anesthetic recovery until meeting discharge criteria.

### Outcomes

The primary outcome was visual clarity, which was assessed subjectively from the surgeon's perspective and objectively via computational image analysis.

### Ethics statement

The study followed the guidelines of the Declaration of Helsinki, approved by the Ethics Committee of the Federal University of Maranhão (CAAE: 32371220.0.0000.5085) on December 17, 2020, and the data collection began after approval by the ethics committee. The study was registered in the Brazilian Registry of Clinical Trials (https://ensaiosclinicos.gov.br/rg/) (RBR-289z7vn) on February 2, 2022. The Free and Informed Consent Form, in writing, was obtained from all patients.

### Statistical treatment

Data were analyzed using SPSS 21.0^®^ (v.22) (EUA). The Shapiro-Wilk test was used to verify normality. The non-parametric Mann-Whitney and chi-square tests were applied to categorical variables to compare the differences in medians between groups. Differences were considered significant when the p<0.05.

## RESULTS

**Figure 1:**
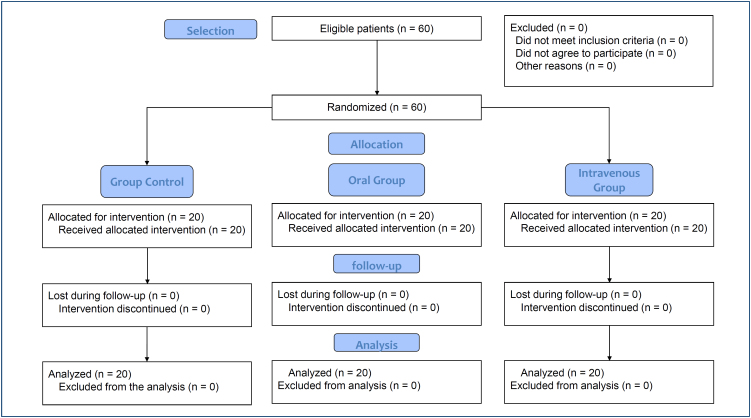
CONSORT flowchart.

In total, 60 patients completed the study: 20 per group (GC, GO, GI). No losses to follow-up occurred (Figure 1).

Female predominance was noted in TXA groups (55%). The median age was 59 years across groups. The GI group had the highest proportion of white participants (75%), and GC had more mixed-race participants (65%). Hypertension was the most common comorbidity (up to 50%).

No significant differences were observed in surgery duration (GC: 185 min [100–295]; GO: 162.5 min [80–250]; GI: 201.5 min [115–290]; p=0.292) or anesthesia time (GC: 255 min [175–415]; GO: 240 min [155–310]; GI: 270 min [170–365]; p=0.097). The number of radiofrequency ablations (p=0.932), pump pressure increases (p=0.419), and total irrigation volume (GC: 43 L [16–85]; GO: 30 L [12–83]; GI: 43 L [17–72]; p=0.119) also did not differ significantly (Table 1).

**Table 1:** Sociodemographic, clinical history, anthropometry, and perioperative data of patients.

Variables		Groups			p-value§
		Control	Oral	Intravenous	
Sociodemographic, n (%)					
Gender					
	Female	9 (45.0)	11 (55.0)	11 (55.0)	0.766
	Male	11 (55.0)	9 (45.0)	9 (45.0)	
Age (years)					
	39–49	4 (20.0)	4 (20.0)	5 (25.0)	0.571
	50–59	7 (35.0)	10 (50.0)	5 (25.0)	
	60 or more	9 (45.0)	6 (30.0)	10 (50.0)	
	Median (Min–max)	59 (39–72)	56 (39–76)	59 (39–75)	0.878£
Race/ethnicity					
	White	7 (35.0)	11 (55.0)	15 (75.0)	0.070
	Black	0 (0.0)	1 (5.0)	0 (0.0)	
	Brown	13 (65.0)	8 (40.0)	5 (25.0)	
Comorbidities					
	SAH^a^	10 (50.0)	10 (50.0)	8 (40.0)	0.765
	DM^b^	3 (15.0)	6 (30.0)	3 (15.0)	0.392
	Dyslipidemia	2 (10.0)	2 (10.0)	6 (30.0)	0.147
	Heart disease	2 (10.0)	3 (15.0)	4 (20.0)	0.676
	Hypothyroidism	1 (5.0)	1 (5.0)	4 (20.0)	0.189
Anthropometry, med (min–max)					
	Weight (Kg)	74 (61–98)	72.5 (59–98)	73.5 (53–98)	0.343
	BMI^c^ (Kg/m^2^)	29.1 (21.4–37.4)	27.8 (23.6–33.2)	28.0 (16.9–35.6)	0.425
Perioperative data, med (min–max)					
	Surgery duration (min)	185 (100–295)	162.5 (80–250)	201.5 (115–290)	0.292
	Anesthesia duration (min)	255 (175–415)	240 (155–310)	270 (170–365)	0.097
	Ablations	4 (0–11)	4.5 (0–8)	3 (0–9)	0.932
	Increases in the pump	0 (0–6)	0 (0–6)	0 (0–7)	0.419
	Irrigation fluid (L)	43 (16–85)	30 (12–83)	43 (17–72)	0.119

Systemic arterial hypertension;

b diabetes mellitus;

c body mass index;

§ chi-square;

£ Kruskal-Wallis. SAH: systemic arterial hypertension; DM: diabetes mellitus; BMI: body mass index.

Subjective clarity scores at first assessment (p=0.415), last assessment (p=0.287), and median scores (GC: 2.8 [2.4–3.0]; GO: 2.8 [2.5–3.0]; GI: 2.9 [2.3–3.0]; p=0.985) showed no group differences. Objective image analysis yielded similar mean clarity values (GC: 1.945 [0.88–4.01]; GO: 1.680 [0.83–3.24]; GI: 1.775 [0.91–3.47]; p=0.421) (Table 2).

**Table 2:** Subjective and objective visual clarity in arthroscopic repair of rotator cuff tears.

Variables		Group			p-value
		Control	Oral	Intravenous	
Subjective visual clarity, n (%)					
First assessment					
	Low	0 (0.0)	0 (0.0)	1 (5.0)	0.415§
	Reasonable	2 (10.0)	3 (15.0)	5 (25.0)	
	Good	18 (90.0)	17 (85.0)	14 (70.0)	
Last assessment					
	Low	0 (0.0)	0 (0.0)	0 (0.0)	0.287§
	Reasonable	2 (10.0)	6 (30.0)	4 (20.0)	
	Good	18 (90.0)	14 (70.0)	16 (80.0)	
	Subjective score med (min–max)	2.8 (2.4–3.0)	2.8 (2.5–3.0)	2.9 (2.3–3.0)	0.985£
Objective visual clarity, med (min–max)					
	Average	1.945 (0.88–4.01)	1.680 (0.83–3.24)	1.775 (0.91–3.47)	0.421£
	Standard deviation	2.135 (0.54–10.91)	2.075 (0.72–4.24)	2.550 (1.02–5.62)	0.599£
	Median	2.240 (1.19–5.46)	2.485 (1.04–4.64)	2.605 (1.27–5.67)	0.559£

£ Kruskal-Wallis;

§ chi-square.

## DISCUSSION

This randomized, double-blind clinical trial included 60 patients who received a placebo, oral, or intravenous TXA. The ­similarity in baseline characteristics between groups suggests that both agents can be used equivalently in arthroscopic rotator cuff repair.

### Studies showing no benefit

Our findings align with several recent studies that found no significant benefit of TXA in improving visual clarity during arthroscopic rotator cuff repair. Nicholson et al.^10^ found no difference in visualization between TXA and CGs (7.2±1.8 vs. 7.4±1.6, p=0.464) using surgeon-completed visual analog scales. Similarly, Bayram et al.^11^ assessed visual clarity in arthroscopic rotator cuff repair and found no difference between epinephrine and TXA groups, with mean VAS scores of 7.6±1.62 versus 7.1±1.74 (p=0.59). Suter et al.^12^ compared intravenous TXA, epinephrine in irrigation fluid, and their combination, finding mean visualization quality scores of 2.1 (±0.40), 2.1 (±0.52), 2.6 (±0.37), and 2.6 (±0.35) for control, TXA, epinephrine, and combination groups, respectively. Regression analysis showed TXA presence/absence did not affect visual clarity, suggesting TXA's hemostatic effect may be insufficient in moderate bleeding scenarios. Yoon et al.^13^ evaluated three TXA administration methods (intravenous, topical, combined) in reverse shoulder arthroplasty and found no differences in hemoglobin drop, blood loss, drain output, or transfusion rates between groups.

### Studies showing benefits

Conversely, several studies demonstrated significant benefits. Takahashi et al.^14^ demonstrated significantly better visual clarity with 1 g TXA, showing a higher percentage of grade 3 visual clarity (75.6±11.2 vs. 68.1±13.4%, p=0.045). Bildik and Pehlivanoglu^15^ investigated intra-articular TXA (250 mg/kg) and found significantly higher visual clarity (1.5±0.5 vs. 2.86±1.7; p<0.001), with a higher percentage of grade 1 clarity (78.1 vs. 32.2%) and lower grade 4 clarity (0 vs. 3.2%). Liu et al.^6^ evaluated 1 g intravenous TXA and found significantly better visual clarity with higher grade 3 scores (53.7±18.9 vs. 40.5±22.1%, p=0.036) and better mean visual scores (2.5±0.2 vs. 2.3±0.3, p=0.048). Ersin et al.^16^ found that visual clarity in the TXA group (10 mg/kg) with mean visual clarity scores=8.1/10 (range=7–10) was significantly better than in the control group (7/10 (range=5–9); p=0.018).

### Analysis of discrepancies

These conflicting results can be explained by several methodological and contextual factors, like: (a) surgical stage specificity, in which Shin et al.^17^ found that 1,000 mg intravenous TXA improved visual clarity during Stage I (synovectomy) compared to control (median grade 1 vs. 2, p=0.027) but showed no effect during acromioplasty, bursectomy, or greater tuberoplasty stages. This suggests TXA's impact is most pronounced during soft-tissue procedures with greater bleeding from the vascular synovial membrane; (b) dosing variability, as Wang et al.^18,19^ demonstrated that combined intravenous plus topical TXA was significantly more effective than either alone, suggesting route and dose optimization may be critical; (c) studies demonstrating benefits may have included higher bleeding risk patients or more extensive procedures. For instance, Pauzenberger et al.^20^ found that 1 g intravenous TXA reduced drain blood, total blood loss, pain, and hematoma formation in total shoulder arthroplasty, suggesting benefits may be more evident in procedures with greater surgical trauma. Also, the (d) assessment methodology, in which studies employed varying scales (3-point, 4-point, VAS 0–10), different assessment frequencies, and both objective and subjective measures. This heterogeneity in outcome measurement may partly explain conflicting results, and (e) procedure complexity and the type and extent of surgical intervention likely influence TXA efficacy. Procedures involving extensive synovectomy or more traumatic tissue manipulation may benefit more from antifibrinolytic therapy than routine rotator cuff repairs.

In summary, our study's absence of global differences in visual clarity aligns with the understanding that TXA benefits may be limited to specific high-bleeding scenarios or vascular procedures, rather than universally beneficial across all arthroscopic shoulder procedures. Standard rotator cuff repair, characterized by relatively controlled bleeding and less extensive synovial work, may not provide the clinical context where TXA's antifibrinolytic properties translate into ­measurable improvements in visual clarity.

### Objective visual clarity

The use of computerized video analysis to objectively quantify visual clarity in this study represents an important methodological advancement in arthroscopic research. While subjective visual analog scales have been the standard for assessing intraoperative bleeding, they are inherently limited by observer variability and lack of standardization.

Recently, Birsel et al.^21^ published a comprehensive validation study of an image processing system for objective quantification of intra-articular bleeding during arthroscopic rotator cuff repair. Their system, which uses color recognition algorithms combining HSV (hue, saturation, value) and CIELAB (International Commission on Illumination of L* for lightness, a* for the red–green axis, and b* for the yellow–blue axis) to calculate bleeding scores based on pixel ratios, demonstrated excellent agreement with experienced surgeon assessments (ICC 0.967, mean absolute error 0.56). Their validation study included 200 video clips evaluated by three senior surgeons, yielding high inter-observer (ICC0.96) and intra-observer (ICC 0.97) reliability. These validation results support the feasibility and reliability of objective visual clarity assessment methods similar to ours. Both approaches recognize that blood in arthroscopy acts like a red dye dissolving into arthroscopic fluid, requiring sophisticated color space analysis beyond simple RGB detection.

### Limitations

This study has several important limitations that should be acknowledged. First, sample size and statistical power. Although our sample size calculation was based on established parameters and previous literature, the study may have been ­underpowered to detect small but potentially clinically relevant differences. Second, measurement bias. The subjective visual clarity assessment, although using a validated three-grade system recorded every 15 min, remains inherently subject to observer bias despite surgeon blinding to patient allocation. While the objective computerized image analysis partially mitigates this limitation, this specific system has not been previously validated in external published studies. Third, procedural variability. Despite standardized surgical technique, inherent variability in aspects such as the number of radiofrequency ablations and adjustments in irrigation pump pressure may independently influence visual clarity beyond the effect of TXA. Fourth, timing and dosing limitations. The study compared specific dosing regimens (20 mg/kg oral, 15 mg/kg IV) and timing protocols (oral 2 h preoperatively, IV 10 min before incision). Different doses, timing strategies, or combined administration routes might yield different results. Finally, procedure-specific considerations. Our findings apply specifically to standard rotator cuff repair in patients with Goutallier grade 0–2 fatty infiltration. Results may not generalize to more complex procedures or massive RCT, where bleeding patterns may differ substantially.

Despite these limitations, this study provides robust evidence from a well-designed, double-blind randomized controlled trial following CONSORT guidelines, with appropriate randomization, concealment, and blinding procedures. The findings contribute important information to clinical decision-making regarding TXA use in arthroscopic rotator cuff repair.

## CONCLUSION

Current evidence suggests that while TXA is safe and well-tolerated, its routine use for visual clarity improvement in arthroscopic rotator cuff repair may not provide universal benefits. The decision to use TXA should be individualized based on patient bleeding risk, procedure complexity, and specific surgical requirements rather than applied universally to all arthroscopic rotator cuff repairs.

## Data Availability

The datasets generated and/or analyzed during the current study are available from the corresponding author upon reasonable request.
